# Supplementary material for: An Assessment of Regional Genetic Diversity of HIV-1
Source: Viruses. 2025 Nov 30;17(12):1568. doi: 10.3390/v17121568 (PMC12737675; doi:10.3390/v17121568)
Supplement: Supplementary file 1 [file viruses-17-01568-s001.zip › viruses-3992429-supplementary.pdf]

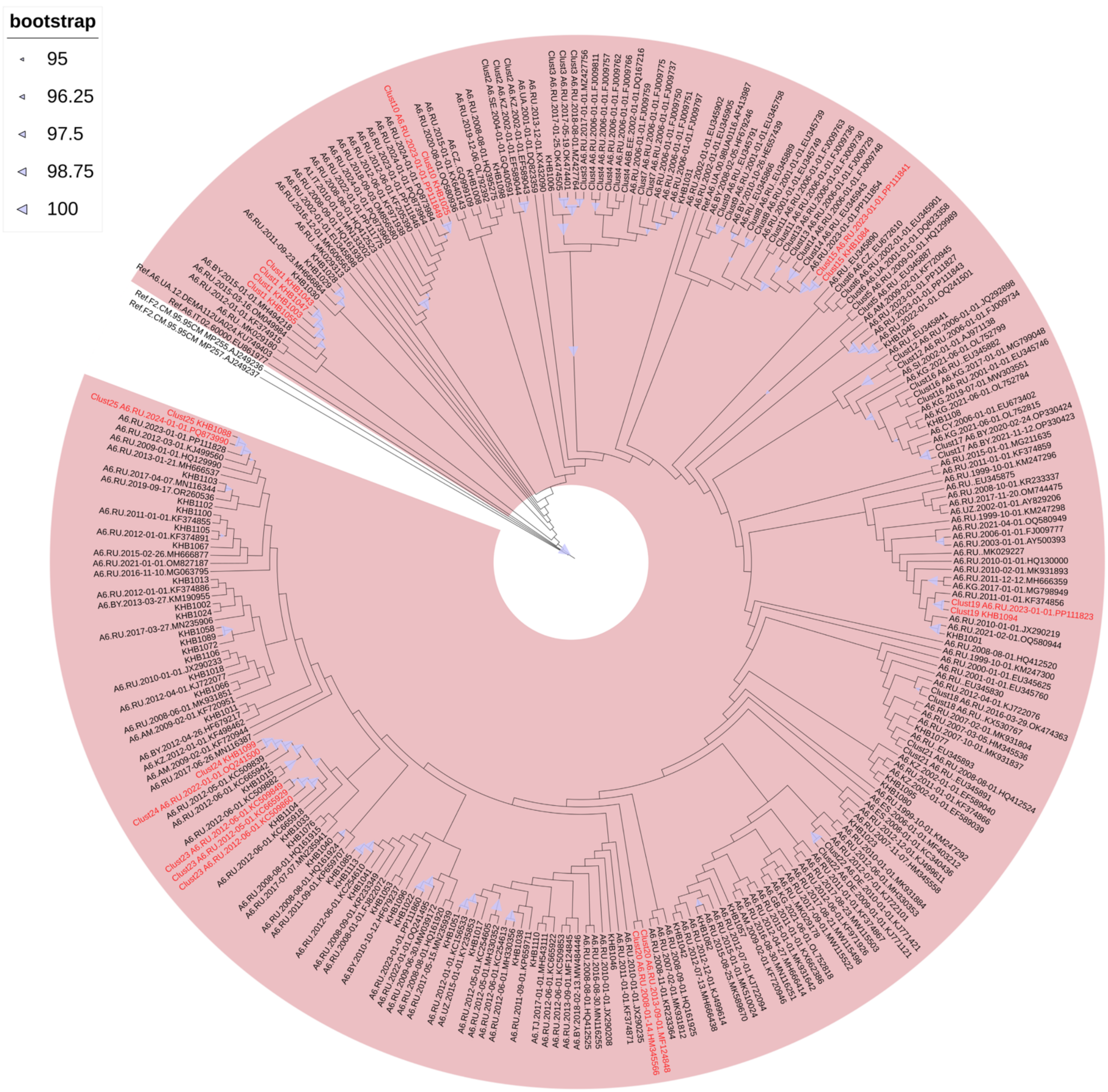

Figure S1. Results of cluster analysis of nucleotide sequences of A6 variant viruses from the Far Eastern Federal District. Clusters containing sequences from the Far Eastern Federal District are marked in red.

Eight clusters containing sequences from the Far Eastern Federal District were identified. Their characteristics are presented in the table below:

Clust1	From Khabarovsk	Sexual transmission (2 male and 2 female)
Clust10	From Khabarovsk	Sexual transmission
Clust15	From Khabarovsk	Injecting drug users
Clust19	From Khabarovsk	Sexual transmission
Clust20	From Yuzhno-Sakhalinsk and Moscow	Presumably, injecting drug users
Clust23	From Khabarovsk	Presumably, sexual transmission (1 male and 2 female)
Clust24	From Khabarovsk	Sexual transmission
Clust25	From Khabarovsk and Vladivostok	Sexual transmission

Clusters were also identified between sub-subtype A6 viruses in other regions of the country (for example, *Clust3, Clust18* – from Noyabrsk (Siberian Federal District) and Krasnodar (Southern Federal District); *Clust7, Clust11, Clust13* – from Saint Petersburg (North-Western Federal District)), as well as in neighboring countries (former Soviet and CIS countries – from Ukraine (*Clust6*) and Estonia (*Clust4*)). This indicates the active circulation of sub-subtype A6 viruses in the country as a whole and in the Far Eastern Federal District in particular, as well as the epidemiological connections with neighboring countries.
